# Relationship between self-care compliance, trust, and satisfaction among hypertensive patients in China

**DOI:** 10.3389/fpubh.2022.1085047

**Published:** 2023-01-18

**Authors:** Chi Zhou, Jingchun Chen, Fang Tan, Sihong Lai, Xu Li, Ke Pu, Jiahui Wu, Yin Dong, Falin Zhao

**Affiliations:** ^1^Department of Health Management, School of Public Health, Hangzhou Normal University, Hangzhou, China; ^2^School of Medicine and Health Management, Tongji Medical College, Huazhong University of Science and Technology, Wuhan, China; ^3^Department of Hospital Office, The People's Hospital of Yuhuan, Taizhou, China

**Keywords:** self-care compliance, trust, satisfaction, hypertensive patients, China

## Abstract

**Introduction:**

Hypertension is a growing public health concern worldwide. It is a leading risk factor for all-cause mortality and may lead to complications such as cardiovascular disease, stroke, and kidney failure. Poor compliance of hypertensive patients is one of the major barriers to controlling high blood pressure. Compliance is not ideal among Chinese patients, and increasing patient self-care compliance with hypertension is necessary.

**Methods:**

This article analyzes the status of self-care compliance, trust, and satisfaction among Chinese hypertensive patients using cross-sectional data from Zhejiang Province. We use a multi-group structural equation model (MGSEM) to compare the interrelationships across genders.

**Results:**

The study's findings show that the average trust, satisfaction, and compliance scores are 3.92 ± 0.55, 3.98 ± 0.61, and 3.33 ± 0.41, respectively. Female patients exhibit higher average total scores for trust and compliance than male patients. The SEM results indicate that trust has a direct positive association with compliance [β = 0.242, 95% CI: (0.068, 0.402)] and satisfaction [β = 0.260, 95% CI: (0.145, 0.367)], while their satisfaction is not directly associated with compliance. The results of MGSEM show that trust has an indirect effect on compliance in the male group through satisfaction [β = 0.051, *P* < 0.05, 95% CI: (0.012, 0.116)]. In the female group, trust has a direct effect on satisfaction [β = 0.235, *P* < 0.05, 95% CI: (0.041, 0.406)] and compliance [β = 0.319, *P* < 0.01, 95% CI: (0.086, 0.574)].

**Discussion:**

This study reveals the mechanisms of self-care compliance, trust, and satisfaction among Chinese hypertensive patients. Its findings may serve as a reference for guiding primary healthcare providers to improve hypertension patients' compliance and implement gender-targeted health interventions.

## 1. Introduction

Hypertension is a crucial public health challenge worldwide (1). It is estimated that the number of people with high blood pressure will reach ~2 billion by 2025 (2–4). Hypertension is the leading risk factor for all-cause mortality, and its complications include cardiovascular disease, stroke, and kidney failure, causing a heavy financial burden on families and society (5–10). In China, ~250 million people suffer from hypertension and increased blood pressure, which may cause 24% of deaths and 14% of disability-adjusted life-years (11). Previous studies have shown that effective self-care management can significantly reduce blood pressure (10–12), such as dietary salt reduction, physical activity, and potassium intake. However, the adoption of self-care behaviors and hypertension control rates are not ideal among Chinese patients (13–15).

As defined by Lahdenpera, self-care compliance means that patients collaborate proactively with healthcare personnel and change their lifestyle according to their recommendations (13, 14). High self-care compliance implies that patients are prone to adopt and maintain a healthy lifestyle to improve blood pressure control (15). In China, most patients with hypertension have low-to-medium levels of self-care compliance (16–19). For example, one study shows that 69.9% of Chinese hypertensive patients have a salt intake higher than 6 g/day, higher than that recommended by the World Health Organization (20). Most studies indicate that hypertension self-care compliance is associated with sociodemographic factors, such as gender, family income, and hypertension duration. However, the literature has devoted limited attention to other potentially influencing factors, such as patients' trust in physicians and patient satisfaction with healthcare services (13, 19).

Trust between doctors and patients are usually defined as the expectation that doctors provide high-quality healthcare services and prioritize patients' interests (21–23). A trusting doctor-patient relationship may increase the communication between patients and doctors, allowing patients to learn valuable medical information and knowledge related to disease management. Doctors may also enhance patients' confidence and vitality and lead them to actively participate in disease management, thus improving patient compliance (5, 24–26). Previous studies have reported that Chinese residents express moderate trust in primary care physicians (27, 28), and female patients are more likely to experience a high level of trust (29). Trust promotes patient satisfaction, and patients with sufficient trust in their physicians are more likely to feel satisfied with healthcare services (30, 31).

Patient satisfaction may be defined as consumption-related fulfillment due to healthcare service and may provide feedback on the technical level and service attitude of medical staff (32–34). Patient satisfaction is a crucial factor in the decision to treat and deliver healthcare services. It is associated with many outcome variables, such as advanced persistence and compliance, effectiveness and efficiency of self-care behavior, and improvement of prognosis (9, 35, 36). A study in China indicates that hypertensive patients have a medium satisfaction score; hence, efforts are needed to improve it (5).

Previous studies of trust, satisfaction, and compliance have focused on the relationship between any two of these factors. For example, a study targeting China has found a significant association between patient trust in their physicians and compliance with diet management and physical activity (2). Another study targeting Lebanon has reported that patients who feel more satisfied with healthcare services have higher hypertension compliance (6). However, quantitative studies that analyze the underlying mechanisms of these three factors among patients with a specific chronic disease are lacking. Exploring these mechanisms is crucial for improving chronic disease management.

To fill this research gap, the current study evaluates the status of patients' self-care compliance, trust, and satisfaction across genders and explores the relationships among patients' self-care compliance, trust, and satisfaction using structural equation modeling (SEM), comparing gender differences.

We hypothesize that (1) trust and satisfaction directly predict self-care compliance, (2) trust directly predicts satisfaction, and (3) satisfaction mediates the role of trust and self-care compliance. Furthermore, due to differences in social and cultural roles, as well as the personality traits and knowledge of hypertension between men and women, we assume that differences may be observed between male and female patients in the above relationships.

## 2. Materials and methods

### 2.1. Participants and design

We conducted this cross-sectional study between June and August 2021. We collected the relevant data using self-administered questionnaires. We addressed two counties (Linping and Chunan) to represent both well- and less-developed GDP levels in Zhejiang Province. Each county featured two medical groups. We chose one hospital and 3–4 of its associated community health centers from each medical group. Thus, we selected four hospitals and 14 community health centers as investigation sites. We recruited residents waiting in the outpatient hall of each hospital/community health center. Participants met the following inclusion criteria: (1) aged 40 years or older, (2) diagnosed with hypertension and receiving antihypertensive therapy, (3) lived in the local area for more than 6 months, and (4) with no cognitive disability. We distributed a total of 450 questionnaires; 373 valid questionnaires were returned, with a response rate of 82.89%.

### 2.2. Measures

#### 2.2.1. Trust

We constructed patient trust using the Wake Forest Physician Trust Scale (WFPTS) designed by Hall et al. (37) and the Chinese version by Dong et al. (28). This scale consists of 10 items and two domains: (1) competence (five items), referring to patients' judgment of physicians' professional knowledge and technical ability, and (2) benevolence (five items), indicating patients' perceived empathy from their physicians or emotional dependence on their physicians. Responses were rated on a five-point Likert scale, ranging from one (“strongly disagree”) to five (“strongly agree”). In contrast, the negatively worded items (items two, three, and seven) were scored in reverse order (“1” = “strongly agree” and “5” = “strongly disagree”). The total score of the C-WFPTS ranges between 10 and 50, with a higher score indicating a higher level of trust in physicians. The reliability and validity of the C-WFPTS are adequate, with a Cronbach's alpha ranging between 0.728 and 0.789 (2, 38), and a Kaiser-Meyer-Olkin (KMO) value of 0.833 (5), suitable for evaluating the trust of Chinese patients in their physicians. The Cronbach's alpha of the scale is 0.827.

#### 2.2.2. Satisfaction

We measured patient satisfaction using a self-developed questionnaire comprising five items: (1) “Are you satisfied with the time spent at treatment and the service process in this hospital?” (2) “Are you satisfied with the technical level and service attitude of medical staff in this hospital?” (3) “Are you satisfied with the burden of medical expenses in this hospital?” (4) “Are you satisfied with the primary health management services (e.g., health consultation, health education, and follow-up services)?” (5) “What about your overall satisfaction in this hospital?” Each question was rated on a five-point Likert scale ranging from one (strongly dissatisfied) to five (strongly satisfied), and the total score ranged from five to 25. Higher scores indicate higher patient satisfaction.

#### 2.2.3. Self-care compliance

We assessed patient self-care compliance using the Compliance of Hypertensive Patients Scale (CHPS) developed by Lahdenpera et al. (14). This scale consists of 13 items and five dimensions: intention (five items), lifestyle (three items), attitude (three items), responsibility (two items), and smoking (one item). Each item was rated on a four-point Likert scale ranging from one (poor compliance) to four (good compliance), and the total score ranged from 13 to 52. Higher scores indicate higher levels of self-care compliance. This scale has been verified as acceptable for measuring hypertension compliance in Chinese patients, with a Cronbach's alpha value of 0.859 (39). In this study, the Cronbach's α for this part is 0.765.

#### 2.2.4. Covariates

Covariates in this study included the socio-demographic characteristics as follows: gender, age, education level (primary school and below, high school or junior college and above), employment status (unemployed or employed), type of medical insurance (medical insurance of urban and rural residents or others, e.g., business insurance), per capita monthly income (≤3,500, 3,501–5,000, or >5,000 RMB), duration of being diagnosed with hypertension (<1, 1–3, 4–6, 7–10, or >10 year), initial medical treatment (community health services centers, hospitals above the county level or clinics and pharmacies), and whether have a family doctor (yes or no).

### 2.3. Statistics analysis

**Figure 1:**
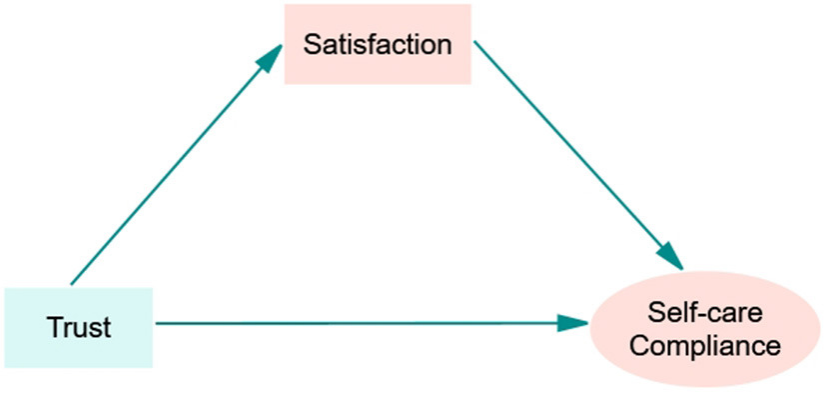
Structural framework of hypothesized relationships.

We entered the data using Epidata 3.1 (the Epidata Association, Odense, Denmark) and analyzed them using IBM SPSS Statistics 26.0 (IBM Corporation, Armonk, NY, USA) and AMOS 24.0 (IBM, New York, NY, USA). First, we employed descriptive statistics to examine participant characteristics and the study's variables. We calculated the proportion and frequencies for categorical data (i.e., gender, district, and education level, among others), and we calculated means and standard deviations (SDs) for quantitative data (i.e., age, score of trust, satisfaction, and self-care compliance). Second, we used a *t*-test to verify the gender differences in trust, satisfaction, and self-care compliance. We employed Spearman's correlation to assess the correlations between the three variables of interest. Third, we used SEM to establish the measurement model outlined in Figure 1. We then used a multi-group structural equation model (MGSEM) to analyze the impact of the proposed variables across genders. We employed the Bootstrap maximum likelihood estimation method to compute the bias-corrected 95% confidence interval (95% CI). To evaluate the model fit, we applied the following criteria: a root mean squared error of approximation (RMSEA) of 0.08 or below, the goodness of fit index (GFI), comparative fit index (CFI), incremental fit index (IFI), and Tucker–Lewis's index (TLI) of 0.90 (40). We set the statistical significance at *P* < 0.05.

## 3. Results

### 3.1. Participants' sociodemographic characteristics

Table 1 reports the sociodemographic characteristics of the 373 participants. The average age of the participants is 66.00 ± 10.41 years old, 55.00% are male, 61.40% have primary school and below education level, and 66.00% have a monthly income of <3,500 RMB. In addition, 44.80% of participants are employed, 72.70% have basic medical insurance for urban and rural residents, 40.50% have suffered from hypertension for more than 10 years, 70.50% chose community health service centers as the initial medical treatment, and 68.1% have a family doctor.

**Table 1:** Sample demographics (*N* = 373).

**Variate**	**Frequency (*N*)**	**Composition ratio (%)**
**Age, mean** **±SD**	66.00 ± 10.41	
**Gender**, ***N*** **(%)**		
Male	205	55.0
Female	168	45.0
**Education level**, ***N*** **(%)**		
Primary school and below	229	61.4
High school	130	34.9
Junior college and above	14	3.8
**Per capita monthly income**, ***N*** **(%)**		
≤ 3,500 RMB	246	66.0
3,501–5,000 RMB	71	19.0
>5,000 RMB	56	15.0
**Employment status**, ***N*** **(%)**		
Unemployed	206	55.2
Employed	167	44.8
**Insurance**, ***N*** **(%)**		
Basic medical insurance for urban and rural residents	271	72.7
Other (business insurance, etc.,)	102	27.3
**Duration of hypertension**, ***N*** **(%)**		
< 1 year	34	9.1
1–3 year	67	18.0
4–6 year	82	22.0
7–10 year	39	10.5
>10 year	151	40.5
**Initial medical treatment**, ***N*** **(%)**		
Community health services centers	263	70.5
Hospitals above the county level	64	17.2
Clinics or pharmacies	46	12.3
**Have a family doctor**, ***N*** **(%)**		
Yes	254	68.1
No	119	31.9

SD, standard deviation.

### 3.2. Descriptive analysis of trust, satisfaction, and compliance scores

Table 2 presents the average scores for each variable. The total average score of the C-WFPTS is 3.92 ± 0.55. The average scores of the competence and benevolence domains are 3.99 ± 0.57 and 3.85 ± 0.64. The total average scores of satisfaction and CHPS are 3.98 ± 0.61 and 3.33 ± 0.41. For the domain scores of CHPS, intention (3.53 ± 0.53) and attitude (3.53 ± 0.52) have the highest average score, while lifestyle (2.76 ± 0.72) has the lowest average score. For the gender subgroup, female patients exhibit higher total average scores for C-WFPTS, and CHPS (*t* = −2.476, *P* = 0.014; *t* = −2.157, *P* = 0.032) than males.

**Table 2:** The description of trust, satisfaction, and self-care compliance scores (mean ± SD).

**Contents**	**Item**	**Average score of each item**			***t*-value**	***P*-value**
		**Total**	**Men**	**Women**		
		**(*****n*** = **373)**	**(*****n*** = **205)**	**(*****n*** = **168)**		
**Trust**	10	3.92 ± 0.55	3.86 ± 0.54	4.00 ± 0.56	−2.476	0.014
Competence	5	3.99 ± 0.57	3.92 ± 0.57	4.08 ± 0.56	−2.709	0.007
Benevolence	5	3.85 ± 0.64	3.80 ± 0.61	3.92 ± 0.67	−1.877	0.061
**Satisfaction**	5	3.98 ± 0.61	4.01 ± 0.61	3.95 ± 0.61	1.011	0.312
**Self-care compliance**	13	3.33 ± 0.41	3.29 ± 0.44	3.38 ± 0.36	−2.157	0.032
Intention	4	3.53 ± 0.53	3.50 ± 0.55	3.57 ± 0.51	−1.144	0.253
Lifestyle	3	2.76 ± 0.72	2.75 ± 0.73	2.77 ± 0.71	−0.227	0.821
Attitude	3	3.53 ± 0.52	3.49 ± 0.55	3.58 ± 0.48	−1.731	0.084
Responsibility	2	3.41 ± 0.72	3.48 ± 0.66	3.32 ± 0.76	2.155	0.032
Smoking	1	3.49 ± 1.04	3.07 ± 1.26	3.99 ± 0.08	−9.427	< 0.001

### 3.3. Correlations between the study variables

Table 3 lists the correlation coefficients between the observed variables. Trust is positively correlated with satisfaction (*r* = 0.249, *P* < 0.01) and self-care compliance (*r* = 0.120, *P* < 0.05). Satisfaction is positively correlated with self-care compliance (*r* = 0.128, *P* < 0.01).

**Table 3:** Correlation coefficients among study variables.

**Variable**	**(1)**	**(2)**	**(3)**
(1) Trust	1.000	-	-
(2) Satisfaction	0.249^**^	1.000	-
(3) Self-care compliance	0.120^*^	0.128^**^	1.000

Significant codes:

** *P* < 0.01;

* *P* < 0.05.

### 3.4. SEM results

**Figure 2:**
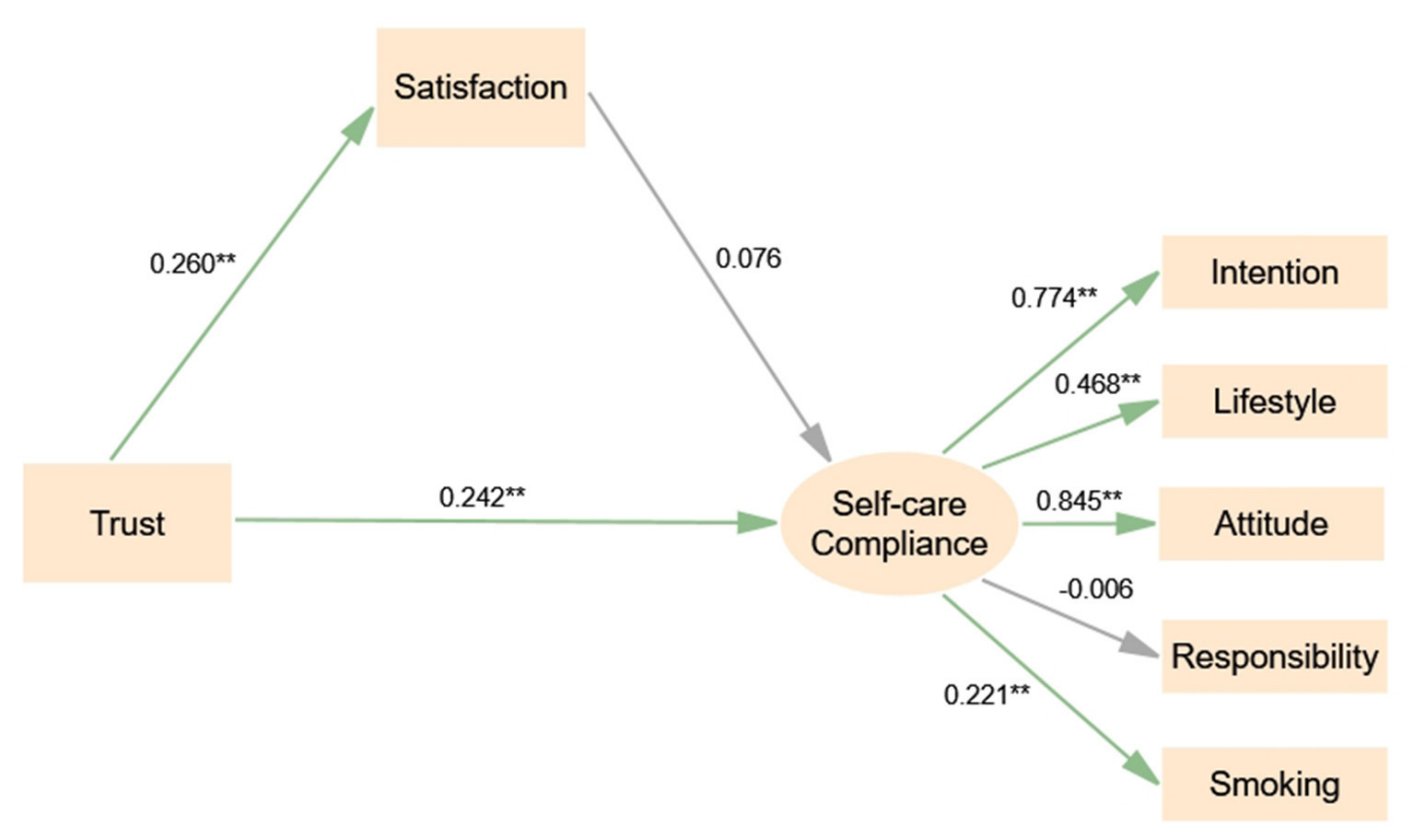
Structural equation model of trust, satisfaction and compliance (full sample). Significant codes: ^**^P < 0.01; ^*^P < 0.05.

The structural model has the following fit indices: chi-square = 2.727, df = 12, *p* < 0.001, CFI = 0.942, GFI = 0.975, IFI = 0.944, TLI = 0.899, and RMSEA = 0.068, indicating a good fit with the data, as shown in Figure 2. The results in Table 4 indicate that trust has a direct positive association with self-care compliance [β = 0.242, *P* < 0.01, 95% CI: (0.068, 0.402)], and trust is positively associated with satisfaction [β = 0.260, *P* < 0.01, 95% CI: (0.145, 0.367)].

**Table 4:** Direct, indirect, and total effects and bias-corrected 95% confidence intervals of the model.

	**Total**				**Male**				**Female**			
	**b**	**SE**	**95% CI**		**b**	**SE**	**95% CI**		**b**	**SE**	**95% CI**	
			**Lower**	**Upper**			**Lower**	**Upper**			**Lower**	**Upper**
**Standardized direct effect**												
Trust → Satisfaction	0.260^**^	0.057	0.145	0.367	0.299^**^	0.075	0.146	0.435	0.235^*^	0.094	0.041	0.406
Trust → Self-care compliance	0.242^**^	0.085	0.068	0.402	0.098	0.106	−0.096	0.313	0.319^**^	0.128	0.086	0.574
Satisfaction → Self-care compliance	0.076	0.061	−0.042	0.199	0.171^*^	0.081	0.022	0.339	−0.023	0.083	−0.173	0.153
**Standardized indirect effect**				
Trust → Satisfaction → Self-care compliance	0.020	0.016	−0.042	0.056	0.051^*^	0.025	0.012	0.116	−0.005	0.022	−0.058	0.035
**Standardized total effect**				
Trust → Self-care compliance	0.262^**^	0.083	0.096	0.420	0.149	0.106	−0.056	0.357	0.313^**^	0.129	0.083	0.571

Standardized estimating of 2,000 bootstrap sample.

SE, standard error; CI, confidence interval.

Significant codes:

** *P* < 0.01;

* *P* < 0.05.

**Figure 3:**
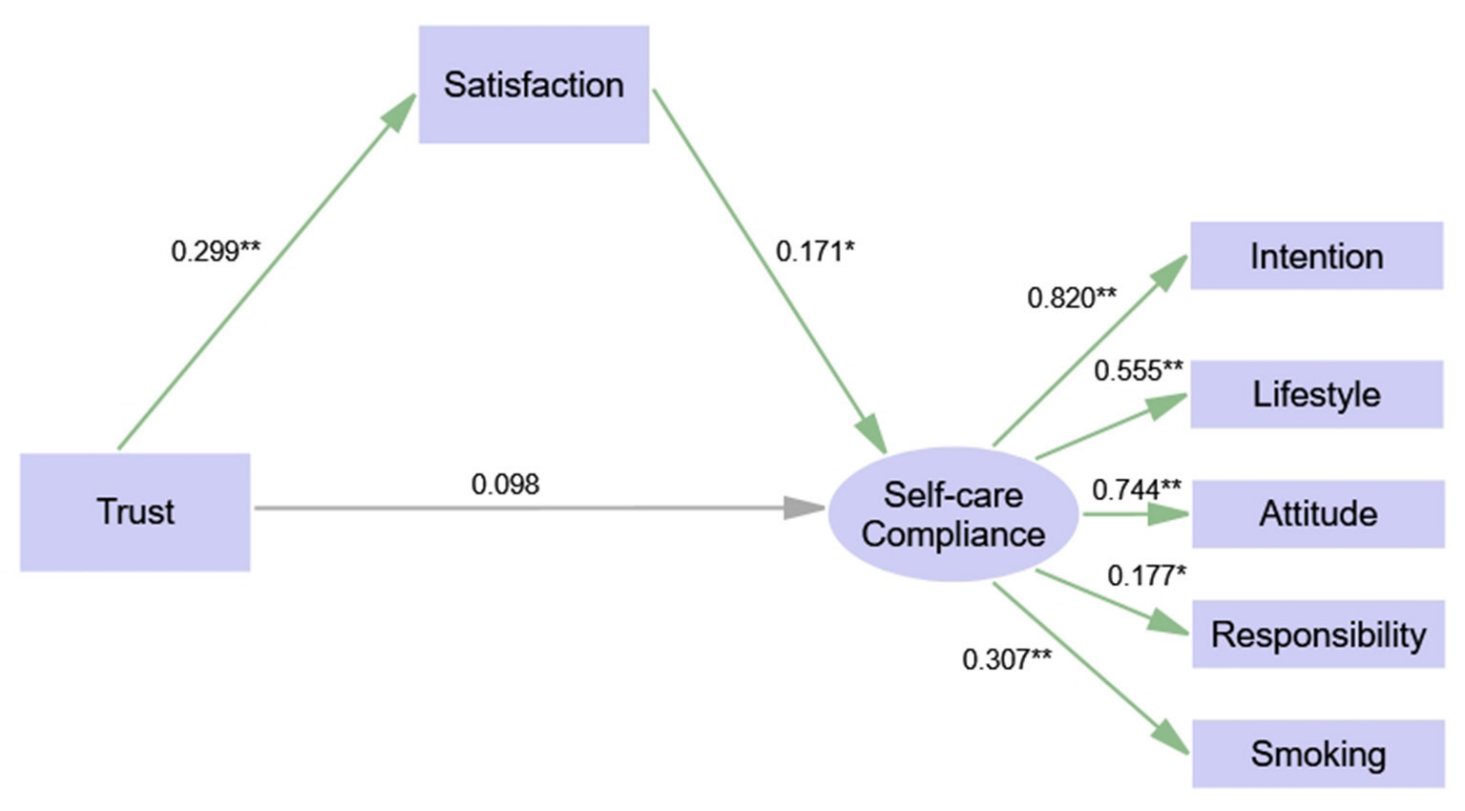
Structural equation model of trust, satisfaction and compliance (sub-sample: male). Significant codes: ^**^P < 0.01; ^*^P < 0.05.

**Figure 4:**
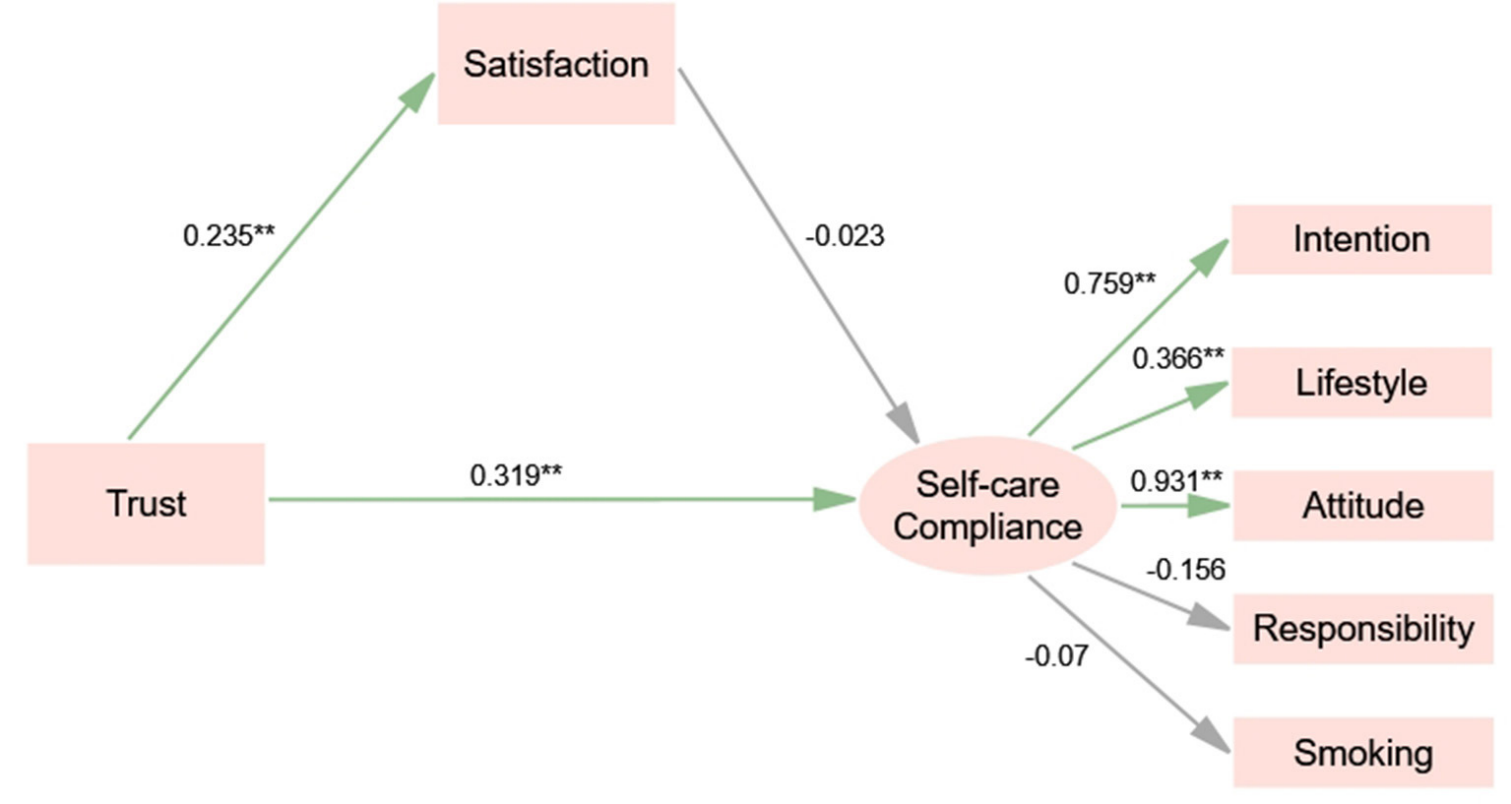
Structural equation model of trust, satisfaction and compliance (sub-sample: female). Significant codes: ^**^P < 0.01; ^*^P < 0.05.

We performed multi-group analyses using SEM by adding constraints ranging from equal structural weights across groups to equal structural weights, covariance, and residuals. The results of the three constraint models show that the chi-square value has no significant correlation with any of the model comparisons (*P* = 0.299, *P* = 0.539, *P* = 0.668), suggesting that the structural model is equivalent in the male and female group. As shown in Figures 3, 4 and Table 4, trust is a direct predictor of satisfaction both in male [β = 0.299, *P* < 0.01, 95% CI: (0.146, 0.435)] and female group [β = 0.235, *P* < 0.05, 95% CI: (0.041, 0.406)]. Satisfaction has a positive direct influence on self-care compliance in the male group [β = 0.171, *P* < 0.05, 95% CI: (0.022–0.339)], while we observe no significant difference in the female group. In addition, trust has an indirect effect on self-care compliance through satisfaction in the male group [β = 0.051, *P* < 0.05, 95% CI: (0.012–0.116)], while it is a direct predictor of self-care compliance in the female group [β = 0.319, *P* < 0.01, 95% CI: (0.086–0.574)].

## 4. Discussion

To the best of our knowledge, this is the first study to employ an SEM model to identify the relationship between trust, satisfaction, and self-care compliance among hypertensive patients, clarify the action mechanisms governing their relationships and compare the results across genders in China. The study's findings provide a reference for improving hypertension compliance.

The study's results indicate that the average CHPS is 3.33 points, in line with a study conducted in Hubei Province in China (39). The highest domain scores of CHPS are intention and attitude, and the lowest domain score is lifestyle. This finding aligns with previous studies (14, 41) and implies that participants may have a positive willingness and attitude to participate in hypertension health management but lack the motivation to take action. Female patients with hypertension have a higher total average CHPS score than male patients, in line with previous findings (41–43). This result may be because, compared with men, women undertake more housework and need to care for family members' health (44). Hence, they have more significant health responsibilities, are prone to higher compliance, and follow a healthier lifestyle (15, 43, 45). In addition, social and cultural factors discourage women from smoking and consuming alcohol in China, which may help maintain a healthy lifestyle (46).

This study finds a total average C-WFPTS score equal to 3.92. This result is slightly higher than the findings from China's western and central provinces (5, 29). One possibility is that the investigated hospital/community health centers in the eastern provinces exhibit higher levels of medical skills training and technology, and medical staff provides better treatment for chronic disease patients, gaining their trust (5, 16, 47). As noted by Chen et al., bridging the gap between the service capacity of primary care institutions and patients' demand for clinical and health services helps build a trustful partnership between doctors and patients (5). However, doctor-patient trust is lower in China than that observed in developed countries (48), especially the benevolence trust level (49, 50). One possible explanation is that the total number of Chinese healthcare professionals is insufficient, and they have a large medical service workload (38). As a result, they spend limited time communicating with patients to pursue service efficiency (2, 23), and patients are often unable to receive enough emotional support from their family doctors (24). Due to the impossibility to increase the number of healthcare professionals in a short time, it might be possible to improve the current situation of physician-patient trust by using text messaging and mobile app (51, 52), which provided a new way to bridge the relationship between physicians and patients. Additionally, this study also shows that female patients exhibit higher trust scores than male patients, in line with a previous study (53). This result may be because women are more sensitive to disease perception (42) and adopt more health-seeking behaviors (54, 55). Therefore, women have more opportunities to communicate with doctors and receive more medical information about their diseases, which helps build a trusted doctor-patient relationship (26, 56).

The SEM results show that trust positively influences self-care compliance among hypertensive patients for the whole sample, in line with existing studies (2, 6, 57). An adequate level of trust leads patients to share more information with doctors about their concerns. Hence, doctors can fully understand patients' attitudes and other potential barriers toward hypertension management and introduce measures for better self-care compliance (45, 58). Moreover, trust is a significant predictor of satisfaction. This result is consistent with the findings of Mahmoudian et al., who show that physicians' emotional and spiritual support, as well as mutual trust between doctors and patients, significantly impact patient satisfaction (24).

Different mechanisms affect the associations between trust, satisfaction, and self-care compliance across genders. The MGSEM results show that satisfaction only significantly affects self-care compliance in the male group. This finding may be due to male patients being more consumeristic in their interactions with the healthcare system and pursuing high-quality health services motivating them to follow up with physicians' instructions (59). In addition, our findings suggest that trust has an indirect positive influence on self-care compliance through satisfaction in the male group, while trust has a direct positive impact on self-care compliance in the female group. This finding confirms the results mentioned above, namely, a higher level of trust leads to better self-care compliance. In addition, compared with men, women are more likely to cognitively trust their doctors. This emotional trust leads them to cooperate with their physicians more actively and have higher compliance in their disease management (42, 60, 61). Men tend to think rationally, and their full trust in their doctors leads them to positively evaluate their treatment outcomes and enhance their motivation to follow doctors' medical advice (62).

Despite its contributions, this study has some limitations. First, the study is only conducted in rural areas in Zhejiang Province of China, which may limit the generalizability of its findings. Second, this cross-sectional study cannot reveal the causal relationship between the variables of interest. Third, we use self-assessed data to verify the research hypotheses. Finally, no objective indicators support the study's findings, which may be subject to answering or memory bias.

## 5. Conclusion

The total scores of patients' self-care compliance, trust, and satisfaction are close to those found by previous studies in China and may still be improved. Female patients have relatively higher self-care compliance and trust than males; hence, special attention should be devoted to male patients in health management. The progress of females' self-care compliance depends on doctor-patient trust, while males' depends on treatment outcome satisfaction through doctor-patient trust. The study's results indicate that gender differences must be considered when developing self-care compliance interventions.

## Data availability statement

The raw data supporting the conclusions of this article will be made available by the authors, without undue reservation.

## Ethics statement

This study was approved by the Hangzhou Normal University Ethics Board (No. 2019065). Written informed consent form was obtained from each participant prior to the enrolment. A copy of the signed consent form was given to each participant.

## Author contributions

CZ and JC: visualization and writing—original draft. YD and FZ: visualization, writing—review and editing, and funding acquisition. FT, SL, XL, KP, and JW: data curation and formal analysis. All authors have read and agreed to the published version of the manuscript.
