# Supplementary material for: Relationship between self-care compliance, trust, and satisfaction among hypertensive patients in China
Source: Front Public Health. 2023 Jan 18;10:1085047. doi: 10.3389/fpubh.2022.1085047 (PMC9889937; doi:10.3389/fpubh.2022.1085047)
Supplement: Supplementary file 1 [file Table_1.pdf]

Supplementary Material

SUPPLEMENTARY TABLE 1 | Differences of the sociodemographic variables between participants from different areas.

Variate	Linping （N=187）	Chunan （N=186）	*P*
**Age, Mean±SD**	66.57±10.24	65.42±10.57	0.290
**Gender, *N*(%)**			0.799
Male	104(55.6%)	101(54.3%)	
Female	83(44.4%)	85(45.7%)	
**Education Level, *N*(%)**			0.679
Primary school and below	111(59.4%)	118(63.4%)	
High school	68(36.4%)	63(33.3%)	
Junior college and above	8(4.3%)	6(3.2%)	
**Per Capita Monthly Income, *N*(%)**			＜0.001
≤3500 RMB	99(52.9%)	147(79.0%)	
3501~5000 RMB	49(26.2%)	22(11.8%)	
＞5000 RMB	39(20.9%)	17(9.1%)	
**Employment Status, *N*(%)**			＜0.001
Unemployed	120（64.2%）	86（46.2%）	
Employed	67（35.8%）	100（53.8%）	
**Insurance, *N*(%)**			0.039
~~Medical insurance of urban and rural residents~~	127（67.9%）	144（77.4%）	
Other (business insurance, etc.,)	60（32.1%）	42（22.6%）	
**Duration of Hypertension, *N*(%)**			0.931
<1 year	17（9.1%）	17（9.1%）	
1~3 year	36（19.3%）	31（16.7%）	
4~6 year	39（20.9%）	43（23.1%）	
7~10 year	18（9.6%）	21（11.3%）	
＞10 year	77（41.2%）	74（39.8%）	
**Initial Medical Treatment, *N*(%)**			0.159
Community health services centers	130（69.5%）	133（71.5%）	
Hospitals above the county level	38（20.3%）	26（14.0%）	
Clinics or Pharmacies	19（10.2%）	27（14.5%）	
**Have a Family Doctor, *N*(%)**			0.235
Yes	122（65.2%）	132（71.0%）	
No	65（34.8%）	54（29.0%）	
